# Strategies for crowdsourcing hearing health information: a comparative study of educational programs and volunteer-based campaigns on Wikimedia

**DOI:** 10.1186/s12889-024-20105-8

**Published:** 2024-09-30

**Authors:** Thais C. Morata, Fernanda Zucki, Adriano Jorge Arrigo, Priscila Carvalho Cruz, Wei Gong, Hector Gabriel Corrale de Matos, Alexandre Alberto Pascotto Montilha, João Alexandre Peschanski, Maria Julia Cardoso, Adriana Bender Moreira Lacerda, Ana Paula Berberian, Eliene Silva Araujo, Débora Luders, Josilene Luciene Duarte, Regina Tangerino de Souza Jacob, Shelly Chadha, Daniel Mietchen, Lane Rasberry, Katia de Freitas Alvarenga, Lilian Cassia Bornia Jacob

**Affiliations:** 1 National Institute for Occupational Safety and Health, Cincinnati, OH United States; 2 Universidade Federal de Santa Catarina, Florianópolis, Brazil; 3 Universidade de São Paulo, Bauru, Brazil; 4 Wiki Movimento Brasil, São Paulo, Brazil; 5 University of Montreal, Montreal, Canada; 6 Universidade Tuiuti do Paraná, Curitiba, Brazil; 7 Federal University of Rio Grande do Norte, Natal, Brazil; 8 Universidade Federal de Sergipe, Aracaju, Brazil; 9 World Health Organization, Geneva, Switzerland; 10 Ronin Institute, Montclair, United States; 11 University of Virginia, Charlottesville, United States

**Keywords:** Science communication, Hearing loss, Hearing health, Educational programs, Wikimedia, Misinformation

## Abstract

**Background:**

Several health institutions developed strategies to improve health content on Wikimedia platforms given their unparalleled reach. The objective of this study was to compare an online volunteer-based Wikimedia outreach campaign with university course Wikipedia assignments (both focused on improving hearing health content in Wikimedia’s public digital knowledge archives), in terms of the reach of the contributions and the extent of the participants’ input. A secondary objective was to examine the feasibility and the implementation of the different strategies.

**Methods:**

The research team partnered for the (1) coordination of improvements in hearing and healthcare content through educational programs using Wikimedia platforms, (2) participation in the global campaign Wiki4WorldHearingDay2023 and (3) evaluation of the proposed strategies. Metrics used in the comparison of the two strategies included the number of articles edited, number of views of the edited articles (as reach) and the extent of edits, captured as the number of words. The feasibility evaluation included assessing recruitment success and the implementation of the proposed plan among faculty, students from various university programs, and volunteers representing different constituencies.

**Results:**

The effort increased the availability of quality plain language information on hearing conditions and hearing care. Both strategies demonstrated to be feasible by their success in recruiting participants who contributed to the effort and by measurable outputs as edits. The contribution of content to Wikimedia platforms as part of education activities provided a more robust result. Wiki4WorldHearingDay2023 145 participants (78 from educational programs) contributed 167,000 words, 258 + references and 140 images to 322 Wikipedia articles (283 existing and 39 new ones), which were viewed 16.5 million times. Contributions occurred in six languages. Edits in Portuguese, mainly by those involved in educational programs, led the number of articles (226 or 70.2%) that were expanded or created during the 5-month tracking period.

**Conclusions:**

The elements that contributed to the success of the studied strategies include an impact topic, coordination with educational programs, international multidisciplinary collaborations, the dissemination of the initiative in several platforms, connection with a robust local Wikimedia affiliate, and the use of a technical infrastructure that provides metrics and coordination mechanisms.

**Graphical abstract:**

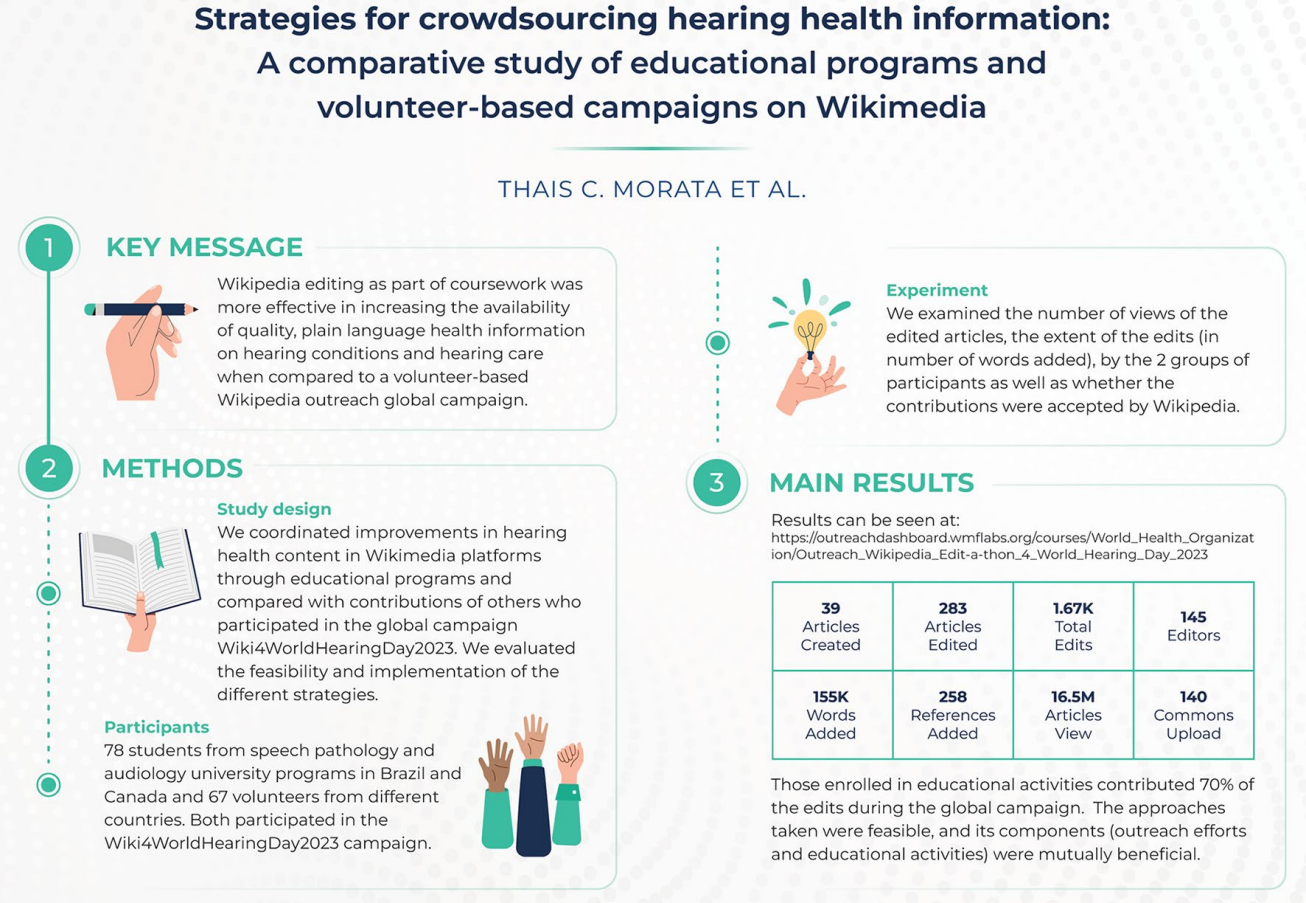

## Introduction

In 2021, the World Health Organization published its first World Report on Hearing, following a resolution from the World Health Assembly of 2017. It highlights not only the individual but also societal costs of hearing loss [1, 2]. It describes how hearing loss can be a seriously disabling condition due to the integral role of hearing in human communication [3]. Hearing-impaired individuals often avoid situations in which communication is difficult, rather than risking a misunderstanding and potentially embarrassing mistakes [2, 4, 5]. This tendency leads to isolation, difficulties at school and work, and possibly adverse psychological consequences [1, 6]. Persons with hearing loss are more prone to falls and accidents as a result of impaired speech perception or an inability to hear audible warnings. Addressing hearing loss has been suggested to be an important modifiable factor toward reducing rates of cognitive decline in populations [1, 2]. Greater awareness and improved health services are needed for the prevention of these hearing disorders [1, 7].

Globally, unaddressed hearing loss was estimated to cost nearly 1 trillion international dollars in 2020, an amount expected to rise unless public health advances are implemented [8]. The World Report on Hearing [2] also contrasts the availability and access to ear and hearing care across the world [2]. As an example, outside high-income countries, it was estimated that only 17% of people who need hearing aids have access to them [9]. There is evidence of a gap of several years between the first signs of hearing difficulties and seeking care [10, 11]. There are many reasons for the gap in ear and hearing care across countries, one of them being the lack of accurate and understandable information [12–14]. The promotion of public health is hindered by the lack of available and accessible information on health conditions and services, a challenge clearly demonstrated for hearing health [14–17].

New communication tools and media facilitate the development and distribution of health information. The challenge of ensuring the material reaches the intended audiences remains. The Pew Research Center reported a trend that social media is taking over traditional information mediums as a primary source of information among Americans [18]. Readers tend to resist information that is pushed onto them, preferring to actively seek out information based on their own needs [19]. That brings up the need to identify where readers are looking for health information as this trend is also seen for individuals seeking public health information. A possible strategy to reach audiences who rely on internet searches is to identify the information pathways they use [18, 19].

Internal metrics from health agencies show that typically their readership is smaller compared to those who seek information on Wikimedia platforms [20, 21]. The Wikimedia Foundation, established in 2003, serves as the umbrella organization for a range of free-knowledge initiatives including but not limited to Wikiversity, Wikidata, and Wikimedia Commons, each with a unique focus and community. Wikipedia is its most widely recognized platform. The Wikimedia Foundation mission centers on empowering individuals globally to create and share knowledge freely [22].

Wikipedia is recognized for advancing public health and is one of the most accessed health information sources [23–26]. Viewership data following the *Ebola crisis* [27] and during the *COVID-19 pandemic* [28, 29] reveals that a significant number of readers located information from authoritative sources such as the World Health Organization and the Centers for Disease Control and Prevention through Wikipedia and related projects, including its media repository Wikimedia Commons and structured data complement, Wikidata. While Wikipedia aims to deliver high quality information with bona-fide citations and links to reliable sources, health organizations have the reverse challenge, as they have difficulty delivering the high quality content they developed to readers at scale [25, 30].

Wikipedia delivers information quickly and effectively to broad, established audiences. Initiatives dedicated to expanding and improving Wikipedia content are also gaining scholarly recognition due to the increasing number of educational programs that incorporate Wikipedia writing assignments [31]. In addition, there is a growing number of peer-reviewed articles on studies that use Wikimedia platforms and datasets in health research, given the accessibility and transparency they provide (see Wikipedia: Academic studies of health information on Wikipedia - Wikipedia).

Science-based information on health conditions, therapies, and events (epidemics or disasters) can be missing/underdeveloped on Wikipedia [32]. Wikipedia’s coverage analysis also revealed lower coverage of issues of interest to non-English nations and gaps in certain areas due to a lack of subject specialist editors [33, 34]. To address this weakness Cochrane [35], Cancer Research UK [36], and the CDC’s National Institute for Occupational Safety and Health [37, 38], are a few of the institutions which developed strategies to integrate content on Wikipedia and evaluate readership/community response. Their efforts align with Open Science principles and other efforts to increase and expedite the reach and accessibility of health information to the public [39, 40].

Some of the strategies previously used by members of the research team and relevant to the present study include (1) organizing small workshops with subject matter experts who work together to improve or create Wikipedia pages on specific topics [41, 42] at either one of the author’s institutions or at scientific conferences and (2) partnering with university graduate, undergraduate, and other training programs, where students contribute subject-specific content to Wikipedia and other Wikimedia platforms [43, 44]. Educational activities offered in the US and Canada are eligible to count with the support, training materials and the metrics-tracking dashboard of the Wiki Education Foundation [45]. For activities conducted abroad (Brazil, South Africa, Sweden, Switzerland) training materials and dashboards are freely available from Programs & Events Dashboard - Meta (wikimedia.org). The activities described in the present study are accounted for in the Wikimedia campaign: Hearing health 2022–2024 [46].

These initial activities served as the basis to a coordinated outreach effort by the National Institute for Occupational Safety and Health (NIOSH) and the WHO Office of Ear and Hearing Care of the World Health Organization. The online effort *Wiki4WorldHearingDay2019* [47] was developed to improve Wikipedia content related to hearing, hearing health services, hearing testing, as well as preventive and rehabilitative interventions [48]. The platform guided participants in creating/editing Wikipedia content and allowed anyone with access to a computer and the internet to participate in the World Hearing Day initiative. This initiative motivated two other efforts, *Wiki4YearOfSound2020* [49] and *Wiki4WorldHearingDay2023* [50] and generated interest in evaluating the strategies that defined it. Following these independent efforts to maximize the availability of focused content on hearing health in Wikimedia’s public digital knowledge archives [45, 51, 52], this project was designed to evaluate specific coordinated activities.

The objective of this exploratory study was to evaluate strategies to maximize the availability of focused content on hearing health in Wikimedia’s public digital knowledge archives. We compared the reach of the edited Wikipedia articles and the extent of contributions from those enrolled in educational activities versus volunteer-based or “non-programmatic” activities which were part of Wiki4WorldHearingDay2023. Secondary objectives included examining the feasibility and the implementation of different strategies.

## Method

The exploratory approach was considered applicable as we aimed to investigate research questions that have not previously been studied in depth. This design is flexible and amenable to using both qualitative and quantitative analysis [53, 54]. It was informed by the principles of the *Reach*,* Effectiveness*,* Adoption*,* Implementation*,* and Maintenance* or RE-AIM framework to guide the planning and evaluation of programs [55] and CDC’s Framework for program evaluation in public health [56]. Public health evaluations have four general purposes: (1) to gain insight when assessing the *feasibility* of an innovative approach to practice, (2) to change practice, (3) to assess effects, and (4) to evaluate the process, which applies at any stage of program development [56]. This study focused on purposes 1 and 4 mentioned above.

The studied initiatives consisted of developing and recruiting participation in the global outreach program Wiki4WorldHearingDay2023 [50] and coordinating improvements of content in hearing and healthcare through University-based educational programs using Wikimedia platforms.

The feasibility evaluation included assessing recruitment success and also involved the adoption/implementation of the proposed plan among faculty, students from various university programs, and volunteers representing different constituencies. These constituencies included the community of Wikipedia editors, researchers affiliated with the proposed institutions, attendees of scientific conferences, and members of NGOs focused on hearing health. The research question on feasibility included: (1) Can interventionists (those who would offer the activity) be recruited? (2) Can they conduct the activity? (3) Can they recruit participants? and (4) Will participants adhere to and engage with the activity? The evaluation of results from each strategy involved the comparison of the contributions from those enrolled in educational activities versus non-programmatic volunteer contributions. Metrics used in the comparison of the two activities included the extent of edits, captured as the number of words. *Reach* was measured by the number of participating institutions, number of Wikipedia edits completed and number of views of the edited content. Edits include minor edits such as spelling corrections, formatting changes, or rearrangement of text without modifying the content and adding, modifying, or improving the actual information in an article. We did not evaluate *maintenance*,* change in practice* or *assessing effects* in the present study. Finally, we decided against examining whether the expansion of the Wikipedia articles completed in the study increased their number of views.

### Participating institutions and coordination

A research consortium composed of professors from seven Brazilian universities (*Universidade de São Paulo; Universidade Federal de Santa Catarina; Universidade Federal do Rio Grande do Norte; Universidade Federal de Sergipe; Faculdade Cásper Líbero; Universidade de São Caetano do Sul; and Universidade Tuiuti do Paraná*), the University of Montreal, Canada, researchers from the Ronin Institute, USA, University of Virginia, USA, the National Institute for Occupational Safety and Health, of the Centers for Disease Control and Prevention, USA, the Ear and Hearing Care Office of the World Health Organization, Switzerland, and *Wiki Movimento Brasil* (a Wikimedia national affiliate), received grant number 2021/06902-2 from the Call for Proposals for Internet Strategic Research [57] from *Fundação de Amparo à Pesquisa do Estado de São Paulo (*FAPESP). In this study we are only reporting the results of two of the grant’s aims. The courses from the six different participating university programs (five undergraduate and one graduate) are identified in Table 1.

**Table 1:** Educational activities in which students contributed to Wiki4WorldHearingDay2023 between November 2022 and April 2023

Educational Programs from which participated in Wiki4WorldHearingDay2023
**Université de Montréal (discipline)** • Promotion and prevention in audiology I and II (Promotion et prévention en audiologie)
**Universidade de São Paulo (disciplines)** • Audiological Theory and Diagnosis II (Teoria e Diagnóstico Audiológico II) • Audiological Theory and Diagnosis III- Pediatrics (Teoria e Diagnóstico Audiológico III – Infantil) • Educational Audiology and Hearing Rehabilitation I (Audiologia Educacional e Reabilitação Auditiva I)
**Universidade Federal de Santa Catarina** • Advanced Topics in Speech Pathology-Language and Audiology Therapy in Primary Health Care (*Tópicos Avançados em Fonoaudiologia na Atenção Primária à Saúde*) (discipline) • Hearing health - Wikipedia as a learning tool in health disciplines (*Saúde auditiva - Wikipédia como ferramenta de aprendizagem em saúde*) (extension activity) • Module XII – The Therapeutic Process I – Therapy of Hearing Disorders (*Módulo XII – O Processo Terapêutico I – Terapia das Alterações da Audição*) (discipline)
**Universidade Tuiuti do Paraná (extension activity)** • Work, Health and Society Study Group (*Núcleo de estudos- Trabalho Saúde e Sociedade*)
**Universidade Federal de Sergipe**,** (extension activity)** • Hearing health - Wikipedia as a learning tool in health disciplines (*Saúde auditiva: Wikipédia como ferramenta de aprendizagem em saúde*)
**Universidade Federal do Rio Grande do Norte (discipline)** • Hearing Development, Assessment, and Intervention (*Desenvolvimento*,* Avaliação e Intervenção em Audição*)

Sources: Campaign: Hearing health 2022–2024 (Articles) [46] / Projeto Saúde Auditiva [58]

The coordination of the educational activities in Brazil was performed by the principal investigator (L.J., *Universidade de São Paulo*), while coordination for the other project activities was done by the first author (T.M., National Institute for Occupational Safety and Health). The coordination and management of the activities are described in a public-facing platform on the Portuguese Wikiversity (*Wikiversidade*,* Projeto Saúde Auditiva*) [58]. Wikiversity is a Wikimedia Foundation project that supports learning communities, their learning materials, and resulting activities.

### Participants

**Fig. 1:**
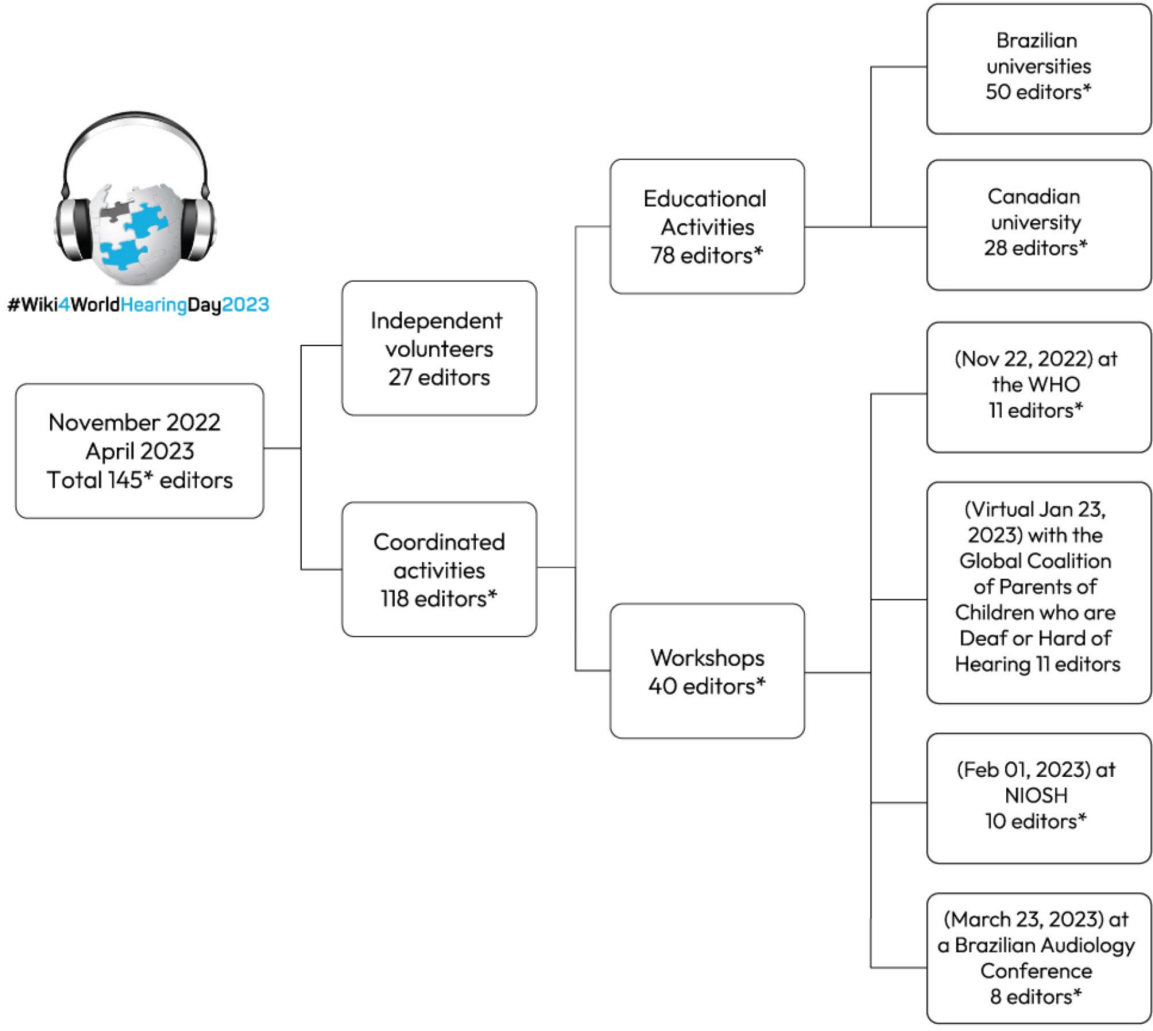
Timeline of activities, activity type, timing and number of contributors to the outreach campaign Wiki4WorldHearingDay 2023 WHO = World Health Organization; NIOSH = National Institute for Occupational Safety and Health; *some of the participants developed content as a group, and only one of the group members published the new content using a single Wikipedia account identifier (username). In these cases, the displayed numbers are undercounts of the total number of participants

One hundred and fifty-nine students who were enrolled in educational activities (either coursework or learning extension programs) between July 2022 and April 2023 were invited to join Wiki4WorldHearingDay2023, 78 registered for it. They can also be considered volunteers as they opted to join and were not paid to do so, but we will refer to them as students to contrast them with the other participants. The remaining 67 Wiki4WorldHearingDay2023 participants were non-programmatic volunteer contributors from different settings, who attended one of the training workshops. Their participation was not associated with any formal educational program. We did not include a separate analysis of the contributions from 27 spontaneous volunteer participants who did not take part of the study’s short training workshops, as it would not have been possible to evaluate how comparable they were to the other participants. Figure 1 displays a timeline of the activities of this research consortium included in this exploratory study.

### Wiki4WorldHearingDay2023 activities

The National Institute for Occupational Safety and Health (NIOSH) has participated in the annual World Hearing Day activities [59] organized by the Office of Ear and Hearing Care of the World Health Organization (WHO) since 2015. Wiki4WorldHearingDay2023 https://meta.wikimedia.org/wiki/Wiki4WorldHearingDay2023 [50] provided a platform (translated into seven languages) and tutorials to guide participants in creating/editing Wikipedia content and allowed anyone with access to a computer and the internet to participate in the campaign. https://outreachdashboard.wmflabs.org/courses/World_Health_Organization/Outreach_Wikipedia_Edit-a-thon_4_World_Hearing_Day_2023 [60].

The authors set up event pages and used many communication channels (partners’ newsletters, emails, social media channels) to invite anyone to register as a participant in Wiki4WorldHearingDay2023 [50]. After registering, participants’ contributions to Wikipedia began to be tracked. Observers can view outcomes for programs in which they are not participants. The dashboard is used by schools of all age levels, universities, edit-a-thons, writing contests and outreach campaigns. We used it to track the progress of both the educational and campaign activities [46]. Note that this link includes other activities not examined by the present study.

As the Program and Events Dashboard [61] tracks every edit done by individuals who joined the program, the authors reviewed edited articles for eligibility to be included and tracked by the outreach dashboard based on their association with the theme of the campaign [60]. The metrics used in this study are described under Tracking of contributions for all participants.

### Short duration workshops

All workshops provided brief information on how to create effective Wikipedia content. The first Wiki4WorldHearingDay2023 hybrid workshop for a group of subject matter experts took place on November 18, 2022 at the World Health Organization headquarters in Geneva. It was attended by approximately 22 participants. Presentations covered the need for a multifaceted approach to improving hearing-related content on Wikipedia and an overview of health knowledge in the Wikimedia ecosystem, including a list of existing Wikipedia articles pertaining to hearing [62]. The WHO streamed and recorded the event, then archived the recording [63]. Participants were from several countries ─ including Chile, Zambia, England, India, Austria, Brazil, Uganda, Australia, Italy, and Georgia.

Toward promoting participation in the workshops for Wiki4WorldHearingDay2023, invitations were distributed by NIOSH and the WHO to their contacts, and a proposal was submitted to hold a 3-hour workshop at an international audiology conference to be held in Brazil. The conference submission was accepted, and it attracted 50 participants. Results were also counted separately and later combined with the main dashboard. Two other small workshops took place, a 4-hour one at NIOSH and a virtual 2-hour one for the Global Coalition of Parents of Children who are Deaf or Hard of Hearing. Two organizations expressed interest in holding similar workshops, but the study group was unable to offer them. Separate dashboards were created to track the different efforts (for which results were counted separately and later combined with the main dashboard). The volunteer participants, like the participants in educational activities, were directed “to improve Wikipedia content related to hearing, hearing health services, hearing testing, preventive and treatment interventions”. However, volunteer participants had greater freedom in selecting articles and topics to work on than those who were enrolled in educational activities. Participants were also encouraged to translate pages from the English Wikipedia into other languages.

Because Wikipedia tracks contributions by account (by username) and because multiple people may have shared a computer and account at the event, we have limited information on whether volunteer Wiki4WorldHearingDay2023 participants (other than those involved in education activities) worked in groups or as individuals.

As the Program and Events Dashboard tracks every edit done by individuals using a username registered in the program, authors have reviewed edited articles for eligibility based on their association with the theme of the campaign. The tracking of unrelated topics was disabled.

### Educational activities

For the duration of the 2-year project (April 2022- March 2024), 159 students from 11 speech pathology and audiology courses at 8 universities were assigned to expand and/or improve Wikipedia articles related to hearing, hearing health services, hearing testing, and hearing preventive and treatment interventions as part of their coursework. Of these, 78 students registered in the Wiki4WorldHearingDay2023 effort and were included in the present study (see Fig. 1). These numbers are likely to be undercounts, as some edits were performed by a group of individuals using a single Wikipedia account identifier.

Instructors and students selected articles for development or expansion based on the subject matter of the class and considering their relevance in the context of each discipline. They also examined related Wikipedia categories (identified at the bottom of each Wikipedia article), such as audiology, acoustics, anatomy, health care, and universal health care.

### Training

A formal training process was developed and implemented throughout the duration of the courses (which ranged from 3 to 12 months). The extent of their edits and participation were used to evaluate the viability of the approach. Instructors reviewed student edits before they were published. As an external quality indicator, we examined whether Wikipedia, through their process of public review, accepted (kept) their edits.

In Brazil, training was offered by experienced Wikipedia writers and editors who used educational materials and tutorials developed by the local Wikimedia affiliate *Wiki Movimento Brasil* [64] and from the training library of the Wikipedia Education Foundation [65]. In Canada, the instructor used the Wiki Edu platform and dashboard, and in-person training was provided by an expert from the local affiliate Wikimedia Canada. The Wikipedia training covered the theoretical framework of the platform; hands-on guidance showed the processes for editing, translating, and developing content. In addition, the Wikipedia Crowdsourcing Group from the University of São Paulo held online workshops and technical support training sessions throughout the effort.

The main objective of the training was to show the students how to discover associated content gaps in Wikipedia, to locate and evaluate sources for their verifiability and to improve the content on the platform. Activities involving Wikipedia included (1) locating and evaluating specific content on the intersection of audiology and hearing care; (2) discussing scientific evidence in these topic areas; (3) engaging in the health education process; and (4) introducing students to a public-directed scientific writing experience.

All courses utilized the Wikipedia-specific “My Sandbox” page, which is a tool offered by Wikipedia that allows students to practice drafting edits in a non-indexed namespace prior to making life changes to Wikipedia. It provides a low-stakes environment to practice editing. Instructors reviewed their edits while they were in “My Sandbox.”

Students had the option to work in groups or individually in 2 of the courses. In 3 extension activities and in 3 courses, students were expected to complete assignments independently; in 2 courses, students were expected to develop content in groups. In all courses, students could select the article to edit based on the course topic and/or from an instructor-provided list, and when they declined deciding, instructors assigned them with a specific topic.

### Tracking of contributions for all participants

The tracking period for edits was from November 17 2022 to March 1 2023. Wikipedia uses the page view statistics tool called *toolforge: pageviews* to report how many people have visited an article in a given time period [66]. The number of views is counted for a month following the end of the activity.

We used Wikimedia’s Program and Events Dashboard [46], a web tool that assists in the management of wiki programs, activities, and events. This web application was adapted from a dashboard created for educational purposes by the Wiki Education Foundation. The platform reports the evolution of interventions and provides statistical parameters that can be used for evaluation [61]. The parameters include the number of page views, number of characters added, number of references added, number of media files incorporated, and indicators of quality of the entry provided by automated tools that use metrics such as readability, amount of information, number of citations/references, syntax, compliance with editing policies and other official website policies [61].

## Results

### Feasibility, adoption and implementation

Our evaluation confirmed the success of recruitment outcomes for each strategy. Faculty and students from various university programs agreed to participate, as did members of the organizations that were offered the opportunity to hold a workshop. They implemented the proposed plan. Each workshop attracted a small number of participants (from 8 to 50). They adhered to and engaged with the activity. Participants did contribute content (editing text, adding citations or media files into Wikipedia). Those who edited outside educational activities were individuals from the community of Wikipedia editors (27 individuals), researchers affiliated with the proponent institutions, attendees of scientific conferences, and members of NGOs focused on hearing health (40 individuals).

### Wiki4WorldHearingDay2023: quantitative assessment of reach

**Fig. 2:**
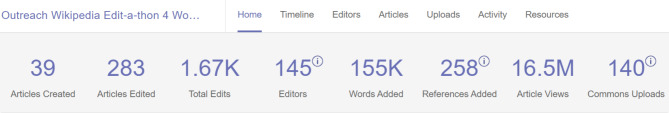
Screenshot from the Program and Events Dashboard [60]

The Dashboard (Fig. 2) displays results indicating the output of the initiative (2 studies strategies plus spontaneous volunteers) during the 5-month tracking period.

One can freely access specific information by clicking the tabs for Timeline, Editors, Articles, Uploads, Activity and Resources and download the data. Separate dashboards were created to track the different efforts (for which results were counted separately and later combined with the main dashboard). Also, information of time and date of an edit allowed us to identify the activity each participant was involved with and compare the outputs of each activity.

At the completion of this effort, edits on topics closely related to hearing and hearing care were classified into the following categories: hearing conditions, anatomical sites, audiological tests, treatments and rehabilitation, health care, risk factors, prevention, musicians’ health, and hearing conditions in film. Contributions occurred in several languages: English, Portuguese, French, Spanish, Chinese, and German. Wikipedia’s content was also expanded on topics that were not only directly related, but also one step removed from target topics, and we kept tracking of those topics. The tracking of unrelated topics was disabled by the authors.

The article that attracted the most views was ChatGPT (12,254,560), a significant outlier. We excluded it from the present analysis but mentioned it here as it illustrates the potential to reach large numbers of readers due to the timeliness of a topic. Content and citations on the challenges and opportunities in the uses of ChatGPT for health services and health communication were added to the ChatGPT Wikipedia article given its relevance to increased availability of online AI resource assistants for health [67], such as S.A.R.A.H. A Smart AI Resource Assistant for Health by the WHO [68] and Alan the Virtual Audiologist in Training [69].

One hundred and ninety-seven articles were edited by participants involved in educational activities, out of 283. The edits completed by those who participated in educational activities were in either Portuguese and French. All edits in French were completed by students. Of 195 articles edited in Portuguese 186 were done by students. Their contributions stand out in the number of articles edited, number of edits (instances when content was added or modified), number of articles created, and number of words added. The time they devoted to the activity was much longer than the time dedicated by the volunteer participants given the nature of their involvement.

Editing Wikipedia was a new type of activity for more than half of the participants of Wiki4WorldHearingDay2023; nonetheless only two of the 39 new articles developed were deleted in the scrutiny of Wikipedia’s public review process [60]. Of the 283 edited Wikipedia articles, 281 were accepted given the quality and appropriateness of the edits and remained in Wikipedia. These were considered as indicators of the success of the effort.

We did not examine whether the expansion of the Wikipedia articles completed in the study increased their number of views. An earlier study examined the fluctuations in the number of views in relation to specific efforts (a video campaign and the launch of a new Wikipedia tool) [70]. The video campaign was not found to be associated with an increase in number of views. The study highlighted methodological challenges, including a series of variables that can affect the outcome. Authors of the present study earlier reported the number of views in a predetermined period before and after former interventions but considered the results to be inconclusive [51].

### Reach and extent of edits from the intersection between Wiki4WorldHearingDay2023 and educational activities

Among the metrics available through Wikimedia’s Program and Events Dashboard we report the number of articles edited, the number of views of the edited articles, the extent of the edits (number of words added), and whether the contributions remained in Wikipedia (deemed acceptable by Wikipedia’s public review process, reported above). Another informative metric is the number of references added to an article. We decided against analyzing the number of references because currently the tool is unable to track when they are added to Wikipedia articles in languages other than English.

The default setting of the tool sets the tracking period for number of views to 30 days after the end of the activity. Caution is needed when interpreting the visualization results. This metric includes the visualization by participants while editing an article. Articles edited and tracked early in the activity have longer tracking periods than those edited on the activity’s last day. However, visualization results provide information on the level of interest from readers across topics.

While all actions can be reviewed in the outreach dashboard [60] and in particular edits by article, by editor in the Articles tab, option “Assessment tools” we selected to report the top five most viewed articles and the top five articles that were more extensively edited, by thematic subcategory (accessed on September 14, 2023). We considered this to be sufficient for the analysis of the strategy results. The resulting number may express the efforts from a single individual or a group (see Table 2). Table 3 displays the five most viewed articles during the Wiki4WorldHearingDay2023, by subtopic and five articles that received the most extensive edits, by subtopic.

**Table 2:** Number of articles edited, number of edits and words added during Wiki4WorldHearingDay2023, by language

Language	# of articles edited/# completed by student participants	total edits	# of articles created	# of words added
English	60/1	120	2	7280
Spanish	10/1	19	1	4900
Portuguese	195/186	575	31/31	103,124
French	9/9	139	0	29,600
German	3	4	0	185
Chinese	6	9	5	13,500
Total	283/197	866	39	1,585,891

Source: Outreach Wikipedia Edit-a-thon 4 World Hearing Day 2023 [60]

**Table 3:** Wiki4WorldHearingDay2023: five most viewed articles and five articles that received most extensive edits, by subtopic. Articles were edited at different times during the campaign, so the tracking duration for each edit was different

Top five most viewed articles by Topic area/articles	Number of views	Number of words added	Top five articles by the extent of edits by Topic area/articles	Number of words added	Number of views
**Hearing Conditions -Total**	**1,298,848**	**876**	**Hearing Conditions -Total**	**30,669**	**5625**
Tinnitus	463,185	545	Trouble du traitement auditif (fr)*	17,293	3164
Meniere’s disease	469,956	36	Pérdida auditiva ocupacional (sp)	3795	1211
Cauliflower ear	187,849	7	Doenças infectocontagiosas e audição (pt)*	3684	264
Cholesteatoma	93,457	288	Hearing loss (zh)	3057	0
Auditory processing disorder	84,401	0 ( ∓ 3)	Perda auditiva induzida por ruído (pt)*	2840	986
**Anatomical Sites - Total**	**238,584**	**72**	**Anatomical Sites - Total**	**7109**	**14,096**
Middle ear	55,635	25	Aparelho vestibular (pt)*	1899	7095
Cochlea	62,816	11	Système auditif (fr)*	1760	1742
Inner ear	46,278	9	Nervo vestibulococlear (pt)*	1498	3577
Ear canal	43,640	9	Sistema auditivo periférico (pt)*	1372	534
Organ of Corti	30,215	18	Órgão de Corti (pt)*	580	1148
**Audiological Tests-Total**	**39,500**	**1992**	**Audiological Tests-Total**	**11,790**	**5584**
Auditory brainstem response	12,122	4	Mascaramento em audiometria (pt)*	2912	1728
Tympanometry	12,810	9	Medida da imitância acústica (pt)*	2842	401
Timpanometria (pt)*	7898	2	Testes acumétricos (deleted-pt)*	2163	0
Audiometria (pt)*	2092	1904	Émission oto-acoustique (fr)*	1969	1363
Electrocochleography	4578	73	Audiometria (pt)*	1904	2092
**Treatment/rehabilitation- Total**	**318,134**	**155**	**Treatment/rehabilitation- Total**	**1587**	**101,485**
Sign language	216,947	4	Leitura labial (pt)*	387	820
*Hearing aid*	86,969	21	Tuba auditiva (pt)*	122	3198
*Tinnitus retraining therapy*	10,289	8	*Hearing aid*	21	86,969
Aparelho de amplificação sonora individual (pt)	731	0	*Tinnitus retraining therapy*	8	10,289
Tuba auditiva (pt)*	3198	122	Habilitação e reabilitação auditiva (pt)*	1049	209
**Health Care- Total**	**268,029**	**772**	**Health Care- Total**	**739**	**192,180**
*Universal health care*	87,729	46	*Primary health care*	277	59,009
*Primary health care*	59,009	277	Global health	201	14,480
COVID-19 vaccine	77,851	82	Sistema Único de Saúde (pt)*	166	28,960
Sistema Único de Saúde (pt)*	28,960	166	Saúde do trabalhador (pt)*	49	2002
Global health	14,480	201	*Universal health care*	46	87,729
**Risk factors- Total**	**287,888**	**146**	**Risk factors- Total**	**13,913**	**4243**
COVID-19 vaccine	256,913	82	Envenenamento por organofosforado (pt)	12,589	400
Poluição sonora (pt)*	4546	14	Ruído ambiental (pt)*	951	477
Pollution sonore (fr)*	5524	14	Efeitos da poluição sonora na saúde (pt)*	39	763
*Ruído (es)*	19,307	14	Ototoxicidade (pt)*	312	1005
Ototoxicite (fr)*	1598	22	Ototoxicite (fr)*	22	1598
**Prevention -Total**	**50,585**	**461**	**Prevention -Total**	**2174**	**11,815**
Ear plug	23,407	9	*Escuta Segura (pt)*	776	380
Health promotion	15,518	69	Safe listening	614	3206
Hearing protection device	6668	53	Dia Mundial da Audição (pt)*	346	266
Lei do silêncio (pt)	3783	10	*World Hearing Day*	320	1209
*World Hearing Day*	1209	320	Construction site safety	118	6754
**Music- Total**	**21,316**	**2434**	**Music- Total**	**2434**	**21,316**
Diplacusis	14,268	235	Riscos auditivos em músicos (pt)*	561	317
*Safe listening*	3206	614	*Health problems of musicians*	248	3145
*Health problems of musicians*	3145	248	Diplacusis	235	14,268
Riscos auditivos em músicos (pt)*	317	561	*Escuta segura (pt)*	776	380
*Escuta segura (pt)*	380	776	*Safe listening*	614	3206
**Hearing Conditions in Film- Total**	**923,631**	**16**	**Hearing Conditions in Film- Total**	**16**	**923,631**
*CODA*	583,354	5	*CODA*	5	583,354
*Sound of metal*	205,384	5	*Sound of metal*	5	205,384
*Children of a lesser God*	102,103	3	*Children of a lesser God*	3	102,103
*List of films featuring deaf and hard of hearing*	32,790	3	*List of films featuring deaf and hard of hearing*	3	32,790

Abbreviations: (pt = Portuguese, fr = French, es = Spanish, zh = Chinese, NA = not available). Asterisks indicate content developed as part of a classroom activity. Italics indicate articles that were both among most viewed and among those that were more extensively edited

For the elaboration of this Table and, consequently, the ranking of the Articles, edits to sandbox users and articles only remotely associated with these themes were excluded. Source: Outreach Wikipedia Edit-a-thon 4 World Hearing Day 2023. [60]

With a few exceptions, Table 3 shows that the most viewed articles were often not among the most edited ones [60]. The subtopic “Hearing conditions” registered the highest number of views (1,298,848), followed closely by those in the category “Hearing conditions in Film” (923,631). The articles that received the most extensive edits were Auditory processing disorder (in French “*Trouble du traitement auditif”)* with 17,293 words added and Organophosphate poisoning (in Portuguese “*Envenenamento por organofosforado”*), with 12,589 words added. Again, with a few exceptions, the articles in Portuguese and French were edited through educational activities.

### Qualitative considerations for the outputs implementation, extent of edits and numbers of views

When conducting qualitative analysis, our focus was to gain a deeper understanding of the specific information sought by those who search Wikipedia for hearing-related content. We also aimed to observe if any intersection between Wiki4WorldHearingDay2023 and educational activities occurred.

Regarding the number of views, it is necessary to consider that English Wikipedia viewership greatly surpasses viewership in other languages. While all categories attracted a large number of viewers during the 5-month tracking period, by far, Hearing Conditions was the category which attracted the most views (1,298,848). In terms of the number of words added to articles in that category, with the exception of the translation to Chinese, most extensive edits happened during educational activities.

Table 3 shows articles in Portuguese and French that were (mostly or exclusively, respectively) edited through educational activities. Several of them were amongst the most viewed (Timpanometria, Audiometria, Tuba auditiva, Sistema Único de Saúde, Poluição sonora, Pollution sonore, Ruído, Ototoxicité, and Riscos auditivos em músicos). Most of the articles which received most extensive edits (in # of words) were also from participants in educational activities.

The category Hearing Conditions in Film also attracted a large number of views (923, 631). The edits to the articles in this category consisted in the inclusion of links to information on hearing care and hearing loss prevention. We consider these fits well with the objective of the awareness raising effort World Hearing Day. We interpreted it as a demonstration of the value of the smallest of edits.

Informal feedback from students and instructors who participated in the present study indicated appreciation for the opportunity to improve their digital literacy and science communication skills, as well as the opportunity to combat misinformation. Faculty and students expressed that they were motivated to participate in order to join the awareness campaign and share knowledge with a wider audience on a publicly accessible platform. The campaign greatly benefited from the participation of those who were involved in the educational programs. Faculty and students reported that, in turn, their participation in Wiki4WorldHearingDay2023 programs provided them with context, inspired them toward the work, and gave them a sense of inclusion and representation.

## Discussion

The objective of this exploratory study was to evaluate strategies to maximize the availability of focused content on hearing health in Wikimedia’s public digital knowledge archives. We compared the reach of the edited Wikipedia articles and the extent of contributions from those enrolled in educational activities versus volunteer-based or “non-programmatic” activities which were part of Wiki4WorldHearingDay2023. Secondary objectives included examining the feasibility and the implementation of different strategies.

Wiki4WorldHearingDay2023 had 145 participants who contributed 167,000 words, more than 258 references and more than 140 images to 322 Wikipedia articles (283 existing and 39 new ones). Both strategies analyzed—educational activities and volunteer-based activities, which were part of Wiki4WorldHearingDay2023—proved to be feasible and mutually beneficial. This was evident from their success in the recruitment of participants and measurable results, such as completed and published edits. Only two of the 39 new articles developed were deleted in the scrutiny of Wikipedia’s public review process. Contributions were made in 6 languages. The participation of those enrolled in education activities had a large impact on the number and extent of contributions completed during the data collection period of this study. Edits in Portuguese, (mainly by those involved in educational programs), led the number of articles (226 or 70.2%) that were expanded or created during the 5-month monitoring period. The number of views gives us a window to which hearing-related topics attract readers. Articles in the category Hearing conditions attracted the most views. This information is valuable to researchers and health and science communicators as it allows them to plan and prioritize their communication efforts.

Previously we have investigated the change in the quality of Wikipedia articles from 2 earlier, similar campaigns, Wiki4WorldHearingDay2019 and Wiki4YearOfSound2020 [51]. Then we used an online tool that uses semantic data to track the evolution of content through article quality assessments. It is based on Objective Revision Evaluation Service (ORES) technology [49, 71], developed and maintained by the Wikimedia Machine Learning team. The average metric for the article quality model indicated that all Wikipedia articles, whether created or edited, improved at a rate ranging from 33–100% [51]. We did not repeat this analysis in the present study.

An extensive body of knowledge exists on a wide range of health outcomes; however, studies from public health settings suggest that evidence-based practices are not disseminated effectively [72–74]. More specifically, we lack data demonstrating that available evidence from the scientific literature is coming to and influencing prevention programs and interventions or reaching the ultimate beneficiary [75]. Wikipedia has been considered an important resource for healthcare information in different contexts, as it is able to reach the public, patients, students, and professionals looking for health-related information online [20, 21, 24].

### Wiki4WorldHearingDay2023: quantitative assessment

The expanded (edited) and new Wikipedia articles in the present study received more than 16.5 million views during the 5-month tracking period. Despite its limitations, the number of article views is a metric often reported when Wikipedia efforts are studied, and it is often impressive [20, 45, 51, 76]. Perhaps due to initial skepticism toward Wikipedia content, the motivation behind reporting this metric was to demonstrate how widely health pages are read by the public, as well as physicians and medical students [21]. Limitations of the number of views metric arise from not being able to exclude the number of views an article receives in the process of it being edited. More significant perhaps is that the period of tracking can be very different in events of long duration. Articles edited and tracked early in the event have longer tracking periods than those edited on the event last day. This is not an issue for short duration events, such as the Vaccine Safety Virtual Wikipedia Edit-a-ton [77].

### Intersection between Wiki4WorldHearingDay2023 and educational activities

Students who were enrolled in this study’s educational activities edited in Portuguese and French contributed the bulk of the new content (70.2%). Edits in other languages were from volunteers who we assumed were not tasked to edit Wikipedia as part of an educational activity. Contributions to Wiki4WorldHearingDay2023 occurred in six languages.

Wikipedia exists in more than 323 official language editions that meet the eligibility requirements established by the Wikimedia Foundation [78]. The content of each Wikipedia edition is independent of the others, but equivalent Wikipedia articles in different languages can be linked via interlanguage links. Wikidata (a collaborative, multilingual, and machine-readable database) is used by various language-editions of Wikipedia to ensure consistency of content across the platform.

### Knowledge equity and health promotion

The Wikimedia Foundation Strategy Process identified a goal to establish a fair and quality representation of knowledge and peoples or “knowledge equity” across the different platforms in the Wikimedia movement [79]. Notwithstanding the editorial freedom that editors enjoy across platforms, there are various community initiatives attempting to coordinate efforts to address various gaps and inconsistencies in content between different languages [80, 81]. We argue that outreach efforts such as those described in the present study contribute to the knowledge equity goal.

In addition, the significance of this effort is magnified by its focus, given the need to increase available and accessible information on hearing health conditions and services, particularly directed to low- and middle-income countries. Expanding the availability of content in Portuguese Wikipedia can prove beneficial not only to populations in Brazil but also to other Portuguese-speaking countries or regions (Portugal, Cape Verde, Angola, Mozambique, Guinea-Bissau, São Tomé and Príncipe, East Timor, Equatorial Guinea, Goa and Macau).

### Comparisons with previous studies

Previous similar efforts included Wiki4WorldHearingDay2019 and Wiki4YearOfSound2020 online programs [51]. These efforts had different durations than the ones included in the present study. Wiki4WorldHearingDay2019 lasted 2 months, while Wiki4YearOfSound2020 lasted 12 months. The Brazilian student participants were involved in an extension activity for which they did not receive a grade or formal credits. Nevertheless, the group edited 37 articles, which attracted more than 220,000 views during the set tracking period. Students were responsible for 60% of the Portuguese-language edits during the Wiki4WorldHearingDay2019 campaign and more than 90% of the Portuguese-language edits during the first half of the Wiki4YearOfSound2020 campaign. Moreover, the quality indexes for pages either created or edited were improved in all situations by registering an increase rate ranging from 33 to 100% [51]. These results are similar to those we report here (from a coursework activity to be graded and a volunteer activity from extension activities), despite the different duration of the events and differences in the type of student participation from the previous study (who were exclusively from an extension activity). The participation of students in extension activities for which they did not receive a grade suggests that the act of sharing knowledge by writing for a larger audience on a publicly accessible platform was enough to motivate participation. Wikipedia education assignments and supervised outreach initiatives generally serve to enhance content curation by promoting digital literacy practices, which can potentially mitigate bias in Wikipedia articles [82]. Wikipedia training resources and public review processes scrutinize new content and sources for their verifiability and neutrality. Specifically, our project involved direct academic supervision of contributions from university programs and campaigns, as well as peer review among contributors who frequently collaborated in groups. These strategies were implemented to reduce the likelihood of content bias.

Participants (students and instructors) who provided spontaneous feedback indicated that the opportunity to combat misinformation was what attracted their participation in the present study. As misinformation in recent years has spread across all areas through different social media, even faster than true and scientific information, the Wikimedia Foundation has adopted strategies to combat misinformation, considering this goal one of its pillars [83, 84]. Previous studies provided a more formal documentation of the reactions from *students* who participated in education programs that include Wikipedia activities [45, 85–91]. A study on the perspectives from *health profession educators* on teaching with Wikipedia reported that two themes summarize the benefits of teaching with Wikipedia: “1) that it provides a meaningful instructional alternative while helping society and developing learners’ information literacy” and “2) that Wikipedia supports learners’ careers and professional identity formation” [92]. Identified challenges included extent of effort and time, restrictive Wikipedia sourcing guidelines, and difficult interactions with stakeholders [92]. For information on the Wikimedia Foundation’s educational efforts, see Wikimedia Communities and Education [93].

### Information gained by number of views versus extent of edits

Students, particularly when new to Wikipedia editing, are often encouraged to contribute to articles that have received a low-quality classification (information usually available for any Wikipedia article). Previous observations indicated that expanding and improving Wikipedia articles is not always associated with an increase in the number of views [24, 25, 38, 51].

Our findings also made two different perspectives and motivations explicit. The views suggest that in the scope of our activities (hearing health), the motivation behind the number of views among the population at large is greater for specific hearing conditions, general information on health care, treatments, and popular portrayals of those in film [24, 25, 38]. This information is valuable for those who work with science and health communication [51]. From the instructors’ and students’ perspective, it was more important to select articles for development or expansion based on the subject matter of the class. Nevertheless, both results (small improvements to articles that attract a large number of edits or more extensive edits to articles not yet attracting a large number of views) contribute to our ultimate objective of increasing the availability of quality plain language health information on hearing conditions and hearing care.

Our experiences in this project can be applied in other contexts. The identification of challenges and opportunities can inform future actions. Our approach is flexible enough to be adapted in a context-specific way for other topics, institutions, and countries. Instructors are not expected to become Wikipedia experts to incorporate Wikipedia activities in the coursework. A total of 141 Wikimedia groups exists and support people around the world to collect and develop educational content under a free license or in the public domain and to disseminate it effectively and globally [94]. The Wikipedia and Education User Group is another source of support, including volunteer members, training materials and tools [95]. In the US and Canada, instructors can use support from Wiki Edu [96].

### Study limitations and recommendations for future actions

While this study satisfactorily met its goals, there were limitations in our approach. The limitations associated with the metric selected for this study were discussed earlier and are inherent to Wikimedia’s Program and Events Dashboard [61]. Some of them are associated with the tracking of activities in Wikipedia in languages other than English. By the time of publication of this article, the number of references added during editing of Wikipedia articles, an important metric, was only tracked by the English Wikipedia. This and other weaknesses are expected to be corrected in the near future.

Another limitation pertains to the different periods for tracking the number of views for editing efforts of long duration. Programs associated with an outreach campaign can address this limitation by shortening its duration. For educational activities, instructors are encouraged to consider asking students to move their edits from the sandbox to Wikipedia’s main space on the same date. Also, if organizers/instructors limited the themes to be edited or preselected specific articles to be expanded or created would make the activity easier to manage and deliver results more closely aligned to their objectives.

We did not include a separate analysis of the contributions from volunteer participants who were not part of formal educational/training activities, as we would not be able to determine how comparable they were to the other participants. Those who attended training events provided meaningful contributions in several languages (English, Portuguese, Spanish and Chinese). The languages included in this activity were based on a convenient sample of participants. For future studies, we recommend comparing the extent and quality of contribution by volunteers who participated in training events and volunteers who did not.

In addition, we did not track or compare contributions by individuals versus the contributions of groups. We suggest studies to determine the effectiveness of each method (individual work with collaborative editing). We also encourage the collection of anonymous data for student feedback.

While a more through evaluation of the contributed content would allow us to further interpret the data from this effort, such analysis would be challenging given the scale of our dataset, and it was outside the scope of the study. However, we are aware that Wikimedia offers tools to facilitate evaluation from this perspective.

While our study demonstrated the feasibility of the approach, it was only feasible after five years of experimenting with different strategies, building of partnerships, an international articulation, the connection with a robust local Wikimedia affiliate and financial support through a grant.

## Conclusion

The crowdsourcing of expertise and knowledge is relevant for public health. The number of views during our effort confirms Wikipedia to be a prominent source of online health information, meriting consideration in health communication and health promotion strategies. This study’s approach can be applied in other contexts. Wikipedia metrics allow researchers to learn what health topics are attracting public interest. The two outreach initiatives described in this study satisfactorily met the goals of this study while increasing the availability of quality plain language health information on hearing conditions and hearing care in several languages. Access to health information can help with decisions to seek care and improve outcomes.

Wikipedia can make a meaningful contribution to improving health literacy in the general population and the working population globally. In addition to increasing the availability and accessibility of science and health content, participants in Wikipedia activities develop academic and digital literacies by learning about sourcing, citing and writing to a new media (encyclopedic) [96]. Participants exercise distinguishing reliable content from misinformation and improve their collaborative skills, particularly online [89].

Finally, the activities described here are aligned with the principles of open science as defined by UNESCO in 2022 [97]. These principles aim to open the processes of scientific knowledge creation, evaluation, and communication to society, making multilingual scientific knowledge openly available, accessible, and reusable. They support the right of everyone to share in scientific advancement and its benefits (Article 27.1 of the Universal Declaration of Human Rights) [98].

## Data Availability

The datasets generated and analyzed during the current study are publicly available at: Campaign: Hearing health 2022–2024 [46]. Outreach Wikipedia Edit-a-thon 4 World Hearing Day 2023 [60]. Projeto Saúde Auditiva [58].

## Declarations

### Disclaimer

The findings and conclusions in this report are those of the author(s) and do not necessarily represent the official position of the National Institute for Occupational Safety and Health, Centers for Disease Control and Prevention. Mention of any company, product or websites external to NIOSH does not constitute endorsement by the National Institute for Occupational Safety and Health (NIOSH), Centers for Disease Control and Prevention. All web addresses referenced in this document were accessible as of the publication date.
